# The Dawning of the Age of Personalized Medicine in Gynecologic Oncology

**DOI:** 10.3390/cancers12113135

**Published:** 2020-10-27

**Authors:** Kimberly K. Leslie, Kristina W. Thiel, Doris M. Benbrook, David Mutch

**Affiliations:** 1 Department of Obstetrics and Gynecology and the Holden Comprehensive Cancer Center, University of Iowa Hospitals & Clinics, Iowa City, IA 52242, USA; 2 Department of Obstetrics and Gynecology, University of Iowa Hospitals and Clinics, Iowa City, IA 52242, USA; Kristina-thiel@uiowa.edu; 3 Stephenson Cancer Center, University of Oklahoma Health Sciences Center, Oklahoma City, OK 73104, USA; doris-benbrook@ouhsc.edu; 4 Division of Gynecologic Oncology, Washington University School of Medicine, St. Louis, MI 63130, USA; mutchd@wustl.edu

It is an undeniable truth that every patient with cancer is unique. So too are the molecular characteristics of every tumor. Yet the standard of care for generations of women with gynecologic malignancies does not account for these complexities and recommends the same treatment for patients whose tumors are broadly categorized based only upon site, stage and grade. In general, the post-surgical treatment modalities employed have changed little over the past decade: for advanced stage cases, the standard treatment is platinum-based chemotherapy and/or radiation for endometrial cancers, platinum-based chemotherapy for ovarian cancers and radiation for high-risk cervical and vulvar cancers.

Until quite recently, molecular agents that target specific pro-growth molecules or pathways have rarely been employed outside of a clinical trial. However, we are now at the beginning of a new paradigm wherein the treatment recommended will be personalized to each case and will involve the use of molecular inhibitors either alone or in addition to standard treatments. We all agree that this is the way forward, but how can we make it happen? This special issue of *Cancers* will explore the current and future opportunities to develop personalized medicine for women with gynecologic cancers with the goals of reporting novel insights and overcoming important barriers to its implementation.

## 1. Hormonal Therapy

Let us begin at the beginning: hormonal therapy. Some may argue that hormonal therapy does not constitute a true personalized treatment, but it is based upon a specific target, hormone receptors, and does meet the criteria for personalized therapy when broadly defined. Progesterone is the natural inhibitor of carcinogenesis in the reproductive tract, and acting through its receptor, PR, is the most important differentiating influence on the epithelia of the Mullerian duct [1]. Progestin therapy has been used in the treatment of endometrial malignant and pre-malignant disorders for many decades and has shown activity against both endometrial and ovarian cancer. More than 70% of patients treated with progestins for atypical endometrial hyperplasia have benefited, and up to 33% of patients with advanced, recurrent endometrial cancer have responded [2]. The clinical efficacy strongly segregates with the expression of hormone receptors in the tumor cells. The Gynecologic Oncology Group (GOG) studied progestins and anti-estrogens in endometrial cancer in studies GOG 81, GOG 121, GOG 81-F, GOG 119, GOG 153 and GOG 168. One of the most active regimens developed today comes from GOG 119 which employed medroxyprogesterone acetate (MPA) on alternating weeks plus tamoxifen continuously [3]. The proposed mechanism for this combination is that tamoxifen, acting as a partial estrogen receptor (ER) agonist and given continuously, induces PRs and ERs and sensitizes cells to the one-week-on, one-week-off MPA. MPA, as a progestin, down-regulates PRs and ERs, so the intermittent dosing allows both receptors to be re-expressed in the off week, thereby sensitizing cells to the next round of MPA therapy. The most predictive biomarker for patient response in GOG 119 was ER expression by immunohistochemistry, indicating that hormone receptor status segregated responders from non-responders [4]. Aromatase inhibitors such as letrozole have also been employed as hormonal therapy with some success in advanced endometrial cancer, particularly when combined with other small molecule inhibitors such as the mTOR inhibitor everolimus [5]. Similarly, the results from 53 trials of different endocrine therapies in epithelial ovarian cancer indicate a clinical benefit of 41% with a trend for a better outcome in cases with ER and PR expression [6]. It is proposed that endocrine therapy may be considered, particularly in low-grade ovarian tumors where ER and PR expression is frequently more robust. At the very least, determining the baseline ER and PR expression profiles of gynecologic cancers should be included in addition to standard pathologic analyses. As with other targeted agents, phase III trials are still needed to confirm the effectiveness of hormonal therapy. It is surprising that despite years of employing hormones for treatment, we still do not have the large clinical trials required to more fully understand the true benefits and appropriate uses of hormonal therapy in gynecologic malignancies.

## 2. Molecularly Targeted Agents

Targeted agents such as tyrosine kinase inhibitors and monoclonal antibody antagonists of angiogenesis and DNA repair have been evaluated in gynecologic oncology clinical trials. Among the single agents deemed active in endometrial cancer are temsirolimus against mTOR (GOG 248), bevacizumab against VEGFA (GOG 229 E) and cediranib (GOG 229 I), a multi-targeted tyrosine kinase receptor inhibitor of VEGFR, PDGFR and FGFR [7,8,9]. For these drugs, the response rate in phase II trials as a single agent was equal to or better than second- or third-line chemotherapy in advanced and recurrent endometrial and ovarian cancer cases. Bevacizumab was shown to improve outcomes in ovarian cancer when it was added to standard chemotherapy, particularly in the highest risk group (ICON7) [10] and when continued as maintenance (GOG 218) [11]. PARP inhibitors such as olaparib have demonstrated activity against serous ovarian cancer when used as maintenance therapy in the SOLO1 [12] trial and in combination with bevacizumab in PAOLA1 [13]. The activity of PARP inhibitors is most striking in the setting of germline or tumor somatic mutations in BRCA1 or 2, but activity is also seen in tumors with other DNA homologous recombination defects [14,15]. Bevacizumab and PARP inhibitors have now entered common clinical use as adjuvant and maintenance therapeutic agents, and this constitutes a significant step forward in the quest to implement personalized medicine in the field. Other targeted agents have been evaluated, as reviewed by Diab et al. [16]. New agents are actively under investigation through the national cooperative group NRG in trials comprising the GY series (GY001–021), and outcomes and results are pending.

## 3. Immunotherapy

The use of immunotherapy in gynecologic malignancies is in the early stages. Thus far, immune checkpoint inhibitors appear to have significant activity, but these agents are most effective against tumors with microsatellite instability [17]. Overall, gynecologic tumors demonstrate response rates of only 10–15% to immunotherapy [18]. In order to achieve greater benefit, ongoing studies are combining immunotherapy with standard treatments and other targeted agents such as tyrosine kinase inhibitors [19]. Our understanding of how effective this strategy will be must await further outcome data from ongoing clinical trials.

## 4. Barriers to the Widespread Deployment of Personalized Medicine in Gynecologic Oncology

While we have demonstrated that therapies targeting molecular tumor characteristics have potential, as described above, a long road remains ahead of us before each patient can benefit from a treatment designed especially for the unique pathological and genomic features of her tumor. We wish to highlight some of the barriers that limit progress in the hopes that with awareness, we will find ways to work together to overcome them. First, it is particularly difficult to translate findings from the laboratory to clinical trials. Independent Principal Investigators in academic institutions typically work through a pharmaceutical company or the NIH to design new clinical trials based upon their findings, but these organizations have their own priorities and mandates. It may take many years to begin, accrue and analyze data from a clinical trial to test new preclinical insights (if a trial is approved at all). We believe that many discoveries from the bench never see the light of day in a clinical trial that could benefit patients. Indeed, one need only review the statistics on the diminished number of interventional trials to see the problem: according to the Society for Gynecologic Oncology, there has been a remarkable 90% reduction in the number of phase III clinical trials in gynecologic oncology from 2011 through 2016 (https://www.healio.com/news/hematology-oncology/20170313/society-outlines-plan-to-address-crisis-in-gynecologic-cancer-trial-access)! Physicians treating women with gynecologic malignancies are held to practicing according to the “standard of care”. Standards are determined by phase III clinical trials. In the absence of such trials, no progress in therapeutics will be forthcoming. Second, the molecular tools required to implement personalized medicine, including tumor genomic sequencing and biomarker evaluation, are often not performed during routine clinical care [20]. One of the major impediments is the reluctance of payors to cover many of these assays, even though they may be standard and non-experimental. Without this information on every tumor, personalized medicine based upon pathologic and/or genomic data cannot be instituted. Third, it is evident that we as basic and translational investigators are just now scratching the surface of the complexity of cancer as a disease, and more support for preclinical studies in gynecologic cancer must be forthcoming to identify the next generation of targeted interventions. Fourth, clinical trials must incorporate translational endpoints in order to identify the biomarkers that predict for response to targeted therapies. It is surprising that many large national and international clinical trials have gone forward without tumor biobanking. Evaluating the pathologic and genomic characteristics of tumors from patients on clinical trials is the necessary first step towards a mechanistic understanding of drug activity and the identification of relevant biomarkers of response. As an example of the power of biobanking and tumor molecular analyses, The Cancer Genome Atlas, instituted and supported by the NCI, has revolutionized our ability to rapidly assess the usefulness of tumor biomarkers and has contributed greatly to our recent progress in personalized medicine [21].

Thus, while we have highlighted some of the barriers that have impeded the implementation of personalized medicine, our goal in this issue is to report on progress and to celebrate the beginning of a new treatment era. We hope you enjoy reading these reports and agree that, at long last, we are at the dawn of the age of targeted molecular therapeutics in gynecologic oncology.
